# Channel Shape Effects on Device Instability of Amorphous Indium–Gallium–Zinc Oxide Thin Film Transistors

**DOI:** 10.3390/mi12010002

**Published:** 2020-12-22

**Authors:** Seung Gi Seo, Seung Jae Yu, Seung Yeob Kim, Jinheon Jeong, Sung Hun Jin

**Affiliations:** Department of Electronic Engineering, Incheon National University, Incheon 406-772, Korea; imyohanzzang@gmail.com (S.G.S.); losnjeo@naver.com (S.J.Y.); scape11@naver.com (S.Y.K.); suytwer3420@naver.com (J.J.)

**Keywords:** InGaZnO, thin-film transistor, instability, channel shape, electric field

## Abstract

Channel shape dependency on device instability for amorphous indium–gallium–zinc oxide (a-IGZO) thin film transistors (TFTs) is investigated by using various channel shape devices along with systematic electrical characterization including DC I-V characeristics and bias temperature stress tests. a-IGZO TFTs with various channel shapes such as zigzag, circular, and U-type channels are implemented and their vertical and lateral electric field stress (E-field) effects are systematically tested and analyzed by using an experimental and modeling study. Source and drain (S/D) electrode asymmetry and vertical E-field effects on device instability are neglibible, whereas the lateral E-field effects significantly affect device instability, particularly for zigzag channel shape, compared to circular and U-type TFTs. Moreover, charge trapping time (*τ*) for zigzag-type a-IGZO TFTs is extracted as 3.8 × 10^4^, which is at least three-times smaller than those of other channel-type a-IGZO TFTs, hinting that local E-field enhancement can critically affect the device reliability. The Technology Computer Aided Design (TCAD) simulation results reveal the locally enhanced E-field at both corner region in the channel in a quantitative mode and its correlation with hemisphere radius (*ρ*) values.

## 1. Introduction

Recently, the requirement on ultra-low power-based driving of thin film transistors has soared due to significant demand on IoT applications, in particular, mobile devices such as smart-phones, smart-watches, and others. Moreover, the newly conceived thin film transistors with high field effect mobility, along with the capability of mass production and full compatibility with the previously setup manufacturing lines, have been welcomed for fulfilling all requirements for the future electronic applications, including flat panel displays [1,2,3]. In this perspective, among a variety of candidates for active materials which could be immediately applied toward the backplanes for flat panel displays (FPDs), amorphous indium–gallium–zinc oxide (a-IGZO) thin film transistors (TFTs) have attracted tremendous attention because they have high current drivability for an ultra-high definition (UHD) flat panel display beyond the advanced a-Si:H TFT technology. Furthermore, high electron mobility (µ_eff_ > 10 cm^2^/Vs), process compatibility with the previously set-up lines, process capability toward large area (>100 inch), high transparency of a-IGZO film are fundamental merits for the future applications of flexible and transparent electronics [4,5,6].

Even though a-IGZO TFTs has been developed to such an extent that they can be applied to commercialized backplanes in flat panel displays such as active-matrix liquid-crystal display (AMLCD) and active-matrix organic light-emitting diode (AMOLED) [7,8], the reliability issues associated with electrical field, temperature, and light induced instabilities are still challenging issues for the mass production of reliable active-matrix flatpanel displays (AMFPDs) based on a-IGZO TFTs [9,10,11,12,13,14]. Among the various parameters that determine the reliability of a-IGZO TFTs, the geometrical shape of the channel for a-IGZO TFTs can be one of the key design parameters to determine the electrical reliability of a-IGZO TFTs [15,16,17,18,19,20]. Up until now, comb-shaped electrodes for amorphous silicon solar cells and interdigitated (e.g., fork-shaped) electrodes in TFTs have been predominantly studied for reduction of gate-to-source capacitance which is one of the key requirements for reduction of kick-back voltage. This is one of the core parameters in determining image quality in FPDs during operation [18]. In addition, ring-shaped (or circular type) electrodes, which are one of the variations in the family of Corbino TFTs, were also studied for the enhancement on insights related to device reliability, which might have the strong dependency on asymmetrical electrical characteristics associated with geometrical configuration on electrodes in TFTs [15,18]. Thus, among a variety of channel shapes, U-type channel for pixel transistors in flat panel display backplanes, do have strong merits for achieving low gate-to-source capacitance (C_gs_) per area, thereby, the U-shape of channel in pixel transistors has been predominately adopted in AMFPD pixel designs, compared with that of the I-type channel [21,22,23]. Beyond reduction of area dependent C_gs_ for pixel transistors, device reliability for TFTs has been one of the long-standing problems in the field of FPDs, even for future TFT-based applications, bio-imaging, X-ray detectors, gas sensors, and others. Until now, even though there were several reports on a-IGZO TFT reliability issues from the perspective of device geometry, interestingly it has been rarely studied for device reliability issues, in particular, channel shapes and their correlation in reliability, which is one of the most critical parameters for determination of electrical performance of a-IGZO TFTs. Furthermore, with only experimental data from implemented TFTs, it is very complicated and picky to identify net effects on device instability, which is purely engaged in device configuration and its geometry driven electric field strength among a variety of processes, materials, and physical dimension issues. The aforementioned parameters are correlated with physical dimension and their materials’ properties. Thus, there is still a lot of room for improvement in understanding of channel geometries and their effects on device reliability via device implementation and electrical measurements, followed by their direct comparison, analytical modeling, their simulation-based analysis, and others.

Herein, we implemented a-IGZO TFTs based on representative channel designs such as: (i) circular type, (ii)zig-zag type, and (iii) U-type. Moreover, device instability characteristics associated with vertical and later electric field effects are evaluated independently, and furthermore, among a variety of factors such as device configuration, channel shapes, physical dimension of TFTs, and others, pure channel shape effects are systematically analyzed with the help of TCAD simulators (SILVACO, Inc.). With this platform, we identified that electric field strength associated with channel shape is highly contributed to device instability. Moreover, the net effects on device instability corresponding to field enhancement factor are quantitatively analyzed through only geometrical variation via TCAD platform, which leads to insight on channel shape and its geometrical variation effects to determine overall device reliability of a-IGZO TFTs. In a word, beyond simple study of academically interesting issues in terms of reliability of TFTs, we expect that this work can be beneficial for the provision of practical guidelines of device design rules in terms of channel shape determination, minimization of parasitic capacitance, and their correlated effects on the enhancement of device reliability in a-IGZO TFTs

## 2. Materials and Methods

**Figure 1:**
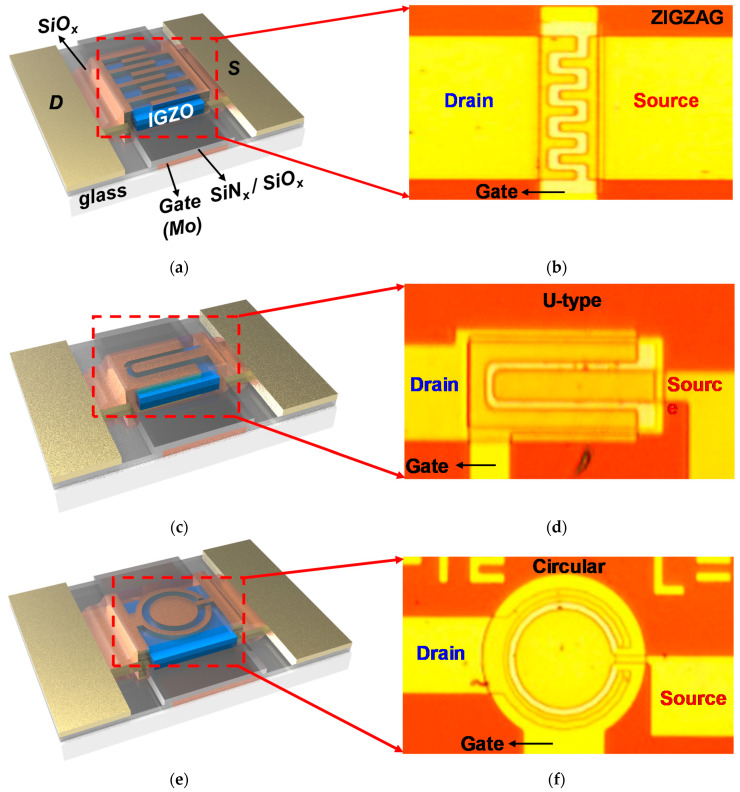
Schematic cartoons for the device configuration corresponding to (**a**) zigzag, (**b**) U-type, and (**c**) circular amorphous indium–gallium–zinc oxide (a-IGZO) thin film transistors (TFTs) and their optical microscope images for the implemented (**d**) zigzag, (**e**) U-type, and (**f**) circular a-IGZO TFTs, respectively.

The a-IGZO TFTs in this study have an inverted-staggered configuration, as shown in Figure 1d. First, 100 nm-thick Mo was deposited by using an RF-sputtering process at 100 W under a chamber pressure of 5 × 10^−3^ Torr in ambient Ar, followed by photo-lithographically patterned and etched in Mo etchant, forming gate electrodes. Thereafter, SiN_x_ (400 nm) were deposited by plasma-enhanced chemical vapor deposition (PECVD), immediately followed by deposition of SiO_x_ (50 nm) in the same PECVD chamber under 300 °C without vacuum break. A 50 nm-thick a-IGZO channel layer was RF-sputtered from IGZO targets (In:Ga:Zn = 1:1:1) for 20 min at 50 W under a chamber pressure of 5 × 10^−3^ Torr in Ar gas. After channel deposition, Mo (70 nm) was deposited and patterned, forming source and drain (S/D) electrodes. For back-channel passivation, a 50 nm-thick SiO_x_ layer was deposited by the PECVD process. Finally contact holes were opened by the reactive ion etching (RIE) process for addressing electrical contact pads.

The channel width and length for all a-IGZO TFTs are 200 and 4 μm, respectively. The electrical characterization of TFTs performance were carried out using Agilent 4155B in a dark and electrically shielded environment. All of the electrical stresses were performed on the thermal chuck at the substrate temperature 60 °C.

## 3. Results and Discussion

### 3.1. S/D Asymmetry Effects for 3-Types a-IGZO TFTs

For understanding of channel shape effects on device reliability for a-IGZO TFTs, transfer characteristics depending on configuration of source and drain (S/D) electrodes were evaluated.

**Figure 2:**
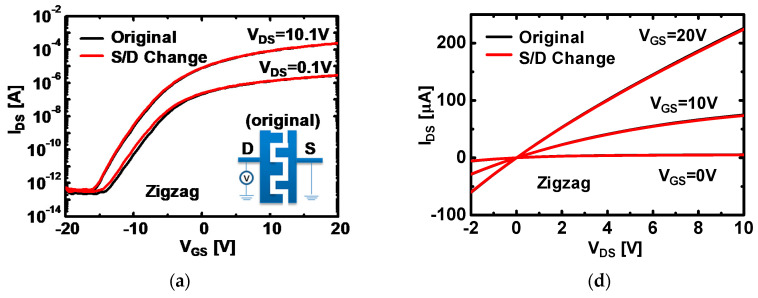

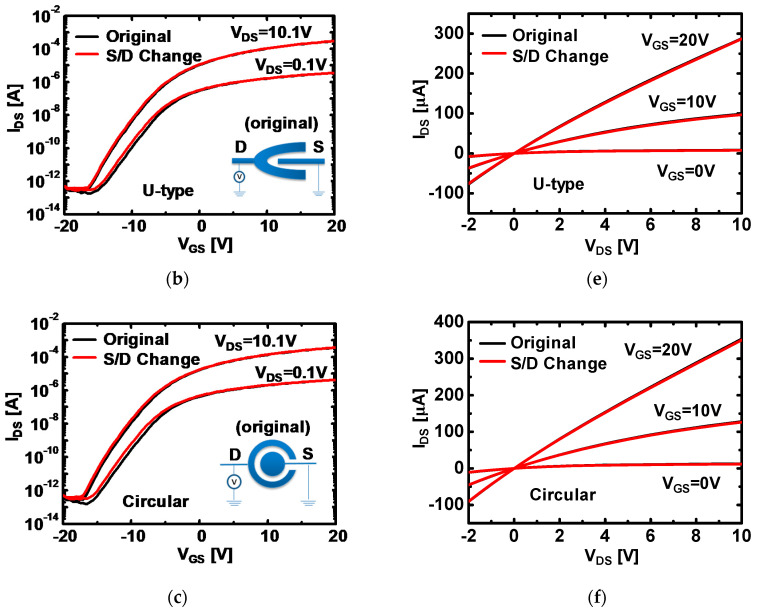
Transfer characteristics of (**a**) zigzag, (**b**) U-type, and (**c**) circular-type a-IGZO TFTs depending on bias configuration. The original is the bias configuration as shown in the insect. Source and drain (S/D) change is a reversed S/D configuration for the same bias value. Output characteristics, corresponding to each channel shape of (**c**) zigzag, (**d**) U-type, and (**e**) circular-type a-IGZO TFTs according to bias configuration. The channel width and length for a-IGZO is 200 and 4 μm, respectively.

Figure 2a–c show the transfer characteristics of 3-types a-IGZO TFTs with two bias configurations: (i) Original and (ii) S/D change. As shown in inset figures, for ‘Original’ configuration, drain bias (V_DS_) was applied to drain electrode, and for ‘S/D change’ configuration, drain bias was applied to the source electrode to investigate the effect of asymmetry in the S/D shape. Transfer characteristics were measured for a different drain bias condition of V_DS_ = 0.1 V and V_DS_ = 10.1 V, respectively. As shown in Figure 2a–c, transfer characteristics were almost identical regardless of channel shape for gate bias (V_GS_) ranging from V_GS_ = −20 to 20 V. The slight deviation in the sub-threshold regime were within experimental error range. Furthermore, output characteristics of 3-types a-IGZO TFTs were measured to confirm asymmetry effects according to variation of V_DS_. The V_GS_ sweeps from −10 to 20 V with 5-V step and output curves at V_GS_ = 20, 10, and 0 V were representatively displayed in Figure 2d–e. The output characteristics of all the devices show decent ohmic properties and negligible difference between the ‘original’ and the ‘S/D change’ configuration. thus, the electrical characteristics were proven to have the same electrical properties irrespective of S/D configuration for all channel shapes. These results indicate that S/D asymmetry effects are negligibly observed, hinting that transfer length (L_T_) in the channel regime is large enough compared with the width of gate electrodes associated with full gate overlapped structures. In addition, electrical parameters for each channel shape were evaluated from transfer characteristics at V_DS_ = 0.1 V. The field-effect mobility for zigzag, U-type, circular a-IGZO TFTs were extracted as 2.77, 4.57, and 5.32 cm^2^/Vs, respectively. Threshold voltage (*V_th_*) based on constant current method [24], which is defined at the drain-to-source current (~ W/L × 10^−8^ A), was extracted as −1.3, −1.9, and −2.8 V for zigzag, U-type, and circular a-IGZO TFTs, respectively. Subthreshold swing and On–off ratio were around 2.6 V/dec and 10^7^ for all devices, respectively. All of the electrical properties for the three device configurations are within similar values except for field effect mobility.

### 3.2. Bias Temperature Stress Instability of 3-Types a-IGZO TFTs

**Figure 3:**
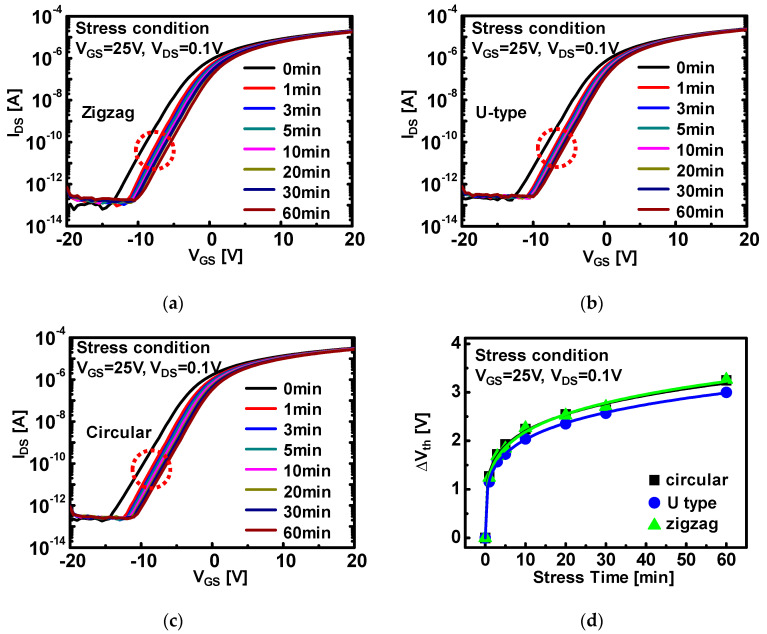
Transfer characteristics of (**a**) zigzag, (**b**) U-type, and (**c**) circular a-IGZO TFTs under bias stress condition of V_GS_ = 25 V and V_DS_ = 0.1 V. All of the bias stress tests were executed under 60 °C substrate temperature for 60 min. Evolutions of threshold voltage shift depending on bias stress time under stress condition of (**d**) V_GS_ = 25 V and V_DS_ = 0.1 V.

For evaluation of device instability depending on channel shape, we performed a bias temperature stress for a-IGZO TFTs with different channel shapes. For monitoring of vertical field effects, V_GS_ of 25 V and V_DS_ of 0.1 V in the linear regime were applied to each device with the substrate temperature of 60 °C for 3600 s. Figure 3a–c shows the evolution of transfer characteristics under bias temperature stress.

After the stress, shifts of *V_th_* and transfer curves for each device were analogous and the variation is within experimental error range, which indicates the vertical field effects on device instability were negligibly observed. The results are thought to have came from the same device configuration of all the devices, particularly for vertical direction. Moreover, without any degradation of electrical properties such as subthreshold swing or on–off ratio, the parallel shift of *V_th_* implies the simple charge trapping between the channel and insulator is associated with device instability. Furthermore, in most of the previous studies of the instability of a-IGZO TFTs under positive bias stress, it has been concluded that electron trapping at the interface and/or in the bulk region of the insulator is the mechanism responsible for a threshold voltage shift (∆*V_th_*) [24,25,26,27,28,29,30,31,32]. In this study, ambient effects were excluded for the origin of bias stress instability by existence of the passivation layer.

For analytical understanding of device instability, the model of ∆*V_th_* by the charge-trapping mechanism was employed in this study. The stretched-exponential equation for the ∆*V_th_* is defined by the following equation [25,33]:

∆*V_th_*(*t*) = ∆*V_th0_* [*1 –* exp {−(*t*/τ)}^β^] (1)

where ∆*V_th0_* is the ∆*V_th_* at infinite time, *τ* = *τ*_o_exp*(E_τ_/kT)* represents the characteristic trapping time of carriers where the thermal activation energy is given by *E_a_ = E_τ_β*. *β* is the stretched-exponential exponent and *E_τ_* is the average effective energy barrier that electrons in the a-IGZO need to overcome before they move into the insulator, and *τ*_0_ is the thermal prefactor for emission over the barrier. Figure 3d shows evolution of ∆*V_th_* (scatter) according to bias stress time for vertical electric field stress (E-field) effect and fitting line from Equation (1). As a result, ∆*V_th_* is well fitted with Equation (1). which can be attributed to the emission of trapped charges toward deep states in the bulk dielectric for longer stress time (*t* > *τ*). Table 1 shows that extracted fitting parameters for a-IGZO TFTs under bias temperature stress in the linear regime. The extracted values for trapping time constant are in the similar range from 1.1 × 10^4^ to 2.8 × 10^4^. Moreover, stretched-exponential fitting parameters (*β*) are about ~0.35. Therefore, these results support the fact that 3-types a-IGZO TFTs, which have exactly the same device structure except for the shape of the S/D electrode, have negligible difference for vertical E-field stress between the channel and insulator.

**Figure 4:**
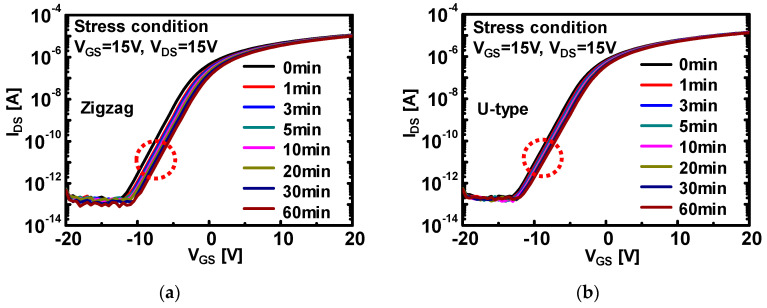

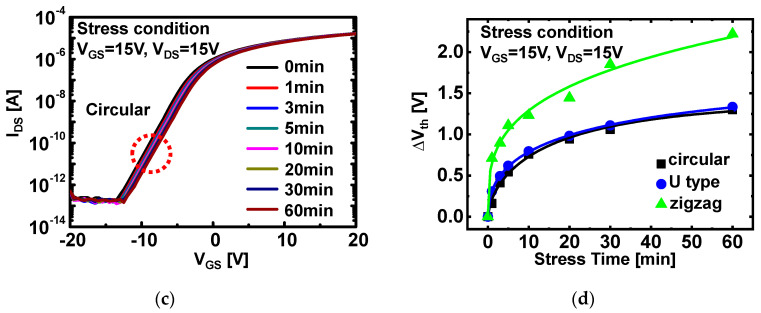
Transfer characteristics of (**a**) zigzag, (**b**) U-type, and (**c**) circular a-IGZO TFTs under bias stress condition of V_GS_ = 15 V and V_DS_ = 15 V. All of the bias stress tests were executed under 60 °C substrate temperature for 60 min. (**d**) Evolutions of threshold voltage shift depending on bias stress time under stress condition of V_GS_ = 15 V and V_DS_ = 15 V.

In parallel, lateral E-field induced instability behaviors for each channel shape were investigated in order to monitor the channel shape dependency on the device instability issue. The V_GS_ of 15 V and V_DS_ of 15 V were applied to each device at the substrate temperature of 60 °C for 3600 s. All of the devices were under saturation regime during the bias temperature stress to enhance the E-field between S/D electrodes. Figure 4a–c display the transfer characteristics under bias temperature stress. Interestingly, zigzag-type a-IGZO TFTs clearly revealed a larger *V_th_* shift, compared to U-type and circular-type a-IGZO TFTs. For clear comparison of ∆*V_th_* depending on channel shapes, ∆*V_th_* versus stress time was evaluated in Figure 4d. The ∆*V_th_* for the zigzag-type device was extracted as 2.1 V, whereas ∆*V_th_* of the other two devices were 1.4 V at 60 min of stress time. It shows the ∆*V_th_* of zigzag-type a-IGZO TFTs have significantly deviated during all the stress time, compared with the other two devices. The distinctive increase of *V_th_* shift was attributed to the strong lateral E-field enhancement associated with geometrical corner of zigzag shape. In the structure of zigzag-type a-IGZO TFTs in Figure 1b, the edge region of the source electrode is surrounded by the drain electrode through three directions, thus, E-field from the drain electrode is anticipated to be focused on the edge region of the source electrode, resulting in the higher magnitude of the E-field than the generally expected value, and local instability in the channel. This makes it possible to explain the phenomenon that the instability (i.e., ∆*V_th_*~1.5 V) was significantly induced in the zigzag-type, compared with those of circular and U-type channel TFTs. The U-type a-IGZO TFTs have one electrode (e.g., drain) which is surrounded by the other electrode (e.g., source). Thus, in the channel region of U-type TFTs, the ratio of the length of straight side to a surrounded channel length between the source and drain and number of corners, together with the curvature of the corner in the channel are critical parameters to determine electric field concentration for the fixed bias voltage. Most regions of the U-type source electrode are closely faced with the drain electrode with only two corners, whereas, in the case of the zigzag-type, it has multiple corners and only edge areas are close to the drain electrode. (As per curvature effects on device instability, it was discussed in the following section) Therefore, U-type a-IGZO TFTs are expected to have relatively less E-field enhancement. As overall comparison for all the bias stress condition and channel shape, ∆*V_th_* and trapping characteristics are summarized in Table 1. Trapping time was much smaller at the stress condition of V_GS_ = 25 V and V_DS_ = 0.1 V for all the devices, leading to the larger ∆*V_th_*. On the other hand, when V_GS_ decreases and V_DS_ increases for stress conditions (V_GS_ = 15 V and V_DS_ = 15 V), ∆*V_th_* was considerably decreased. Considering the results from Table 1, vertical E-field more strongly affects ∆*V_th_* than lateral E-field. In addition, U-type a-IGZO TFTs exhibit relatively better stability for bias temperature stress among devices. As adopted for a-Si TFTs, U-type is known to have lower gate-to-source overlapped capacitance (C_GS_), thus, this result is well matched with geometrical channel shape effect for electrical instability [23].

### 3.3. Study on E-Field Distribution in the Channel Depending on Channel Shape

**Figure 5:**
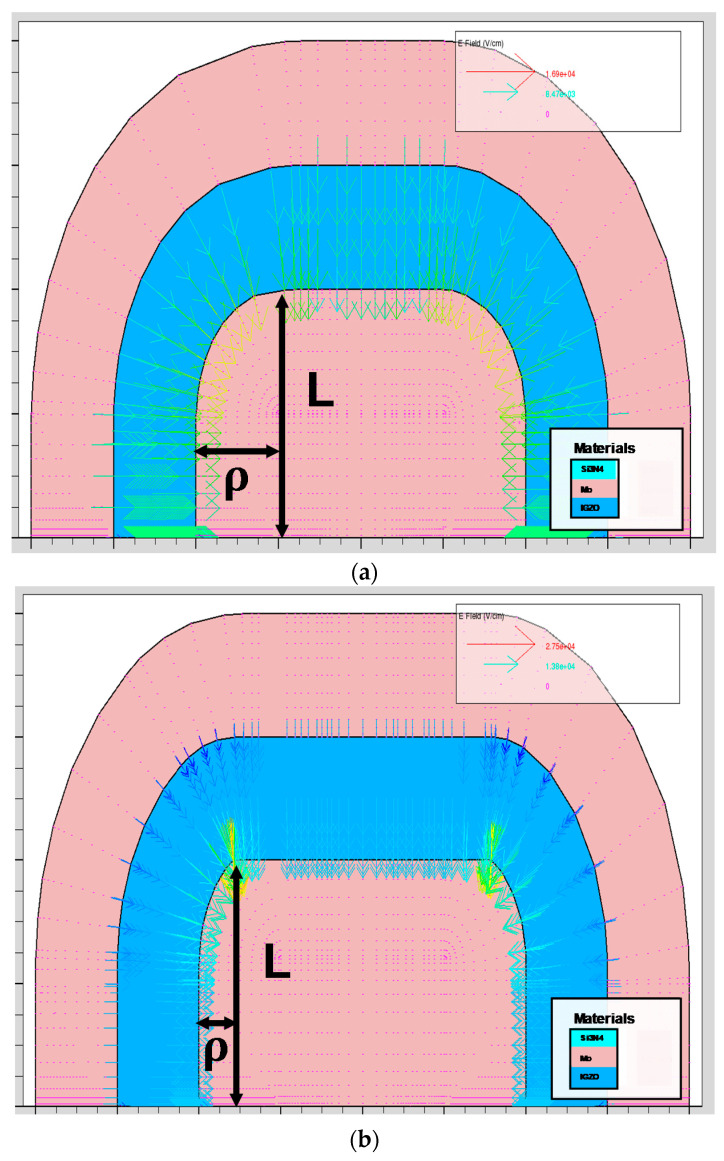

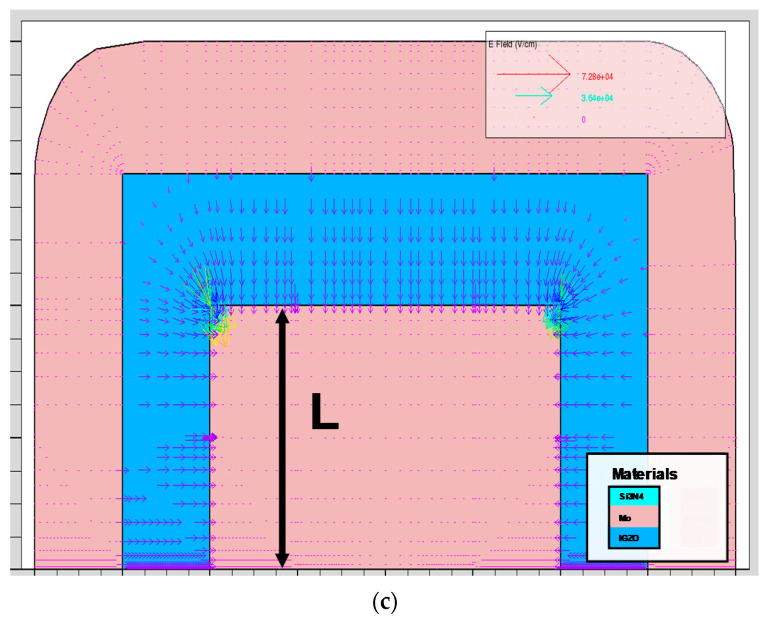
Simulated electric field stress (E-field) distribution from device structures with (**a**) larger *ρ* (round corner), (**b**) small *ρ*, and (**c**) *ρ* = 0 (perpendicular corner). The distribution of E-field was focused and the magnitude was enhanced at the corner region of the channel, especially for devices with a smaller *ρ*.

**Figure 6:**
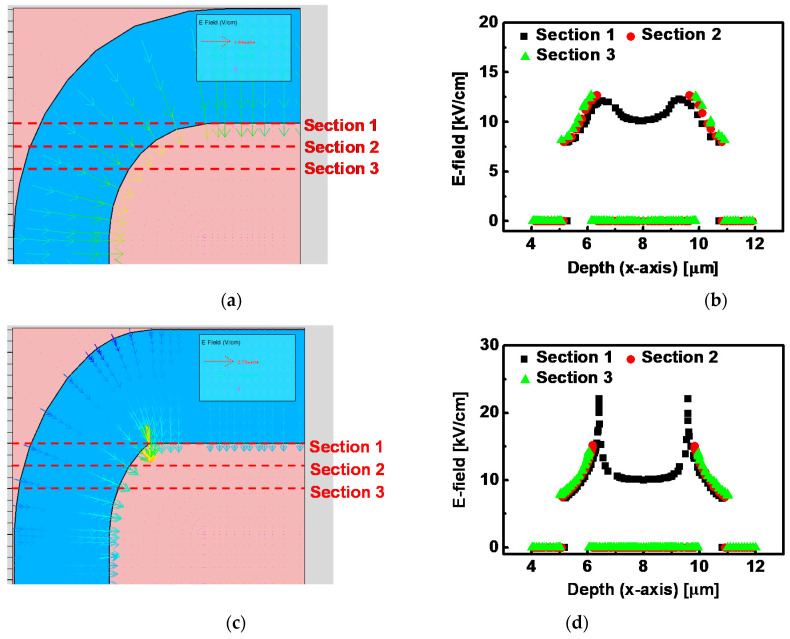

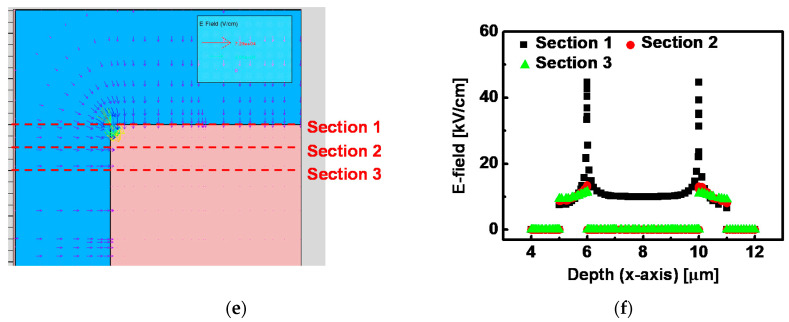
Enlarged device structure and extracted E-field distribution near the corner region with (**a**,**b**) larger *ρ* (round corner), (**c**,**d**) small *ρ*, and (**e**,**f**) *ρ* = 0 (perpendicular corner).

**Figure 7:**
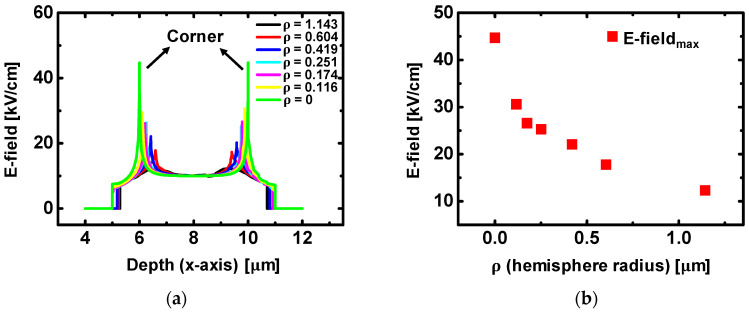
(**a**) E-field variation at Section 1 depending on hemisphere radius (*ρ*) of the channel. (**b**) Extracted maximum E-field values at the different hemisphere radius.

For the better understanding of E-field enhancement depending on channel shape, Technology Computer Aided Design (TCAD, Silvaco Inc.) simulation was employed to analyze E-field distribution in the channel in a quantitative mode [34]. Figure 5a–c shows simulated results with device structures depending on different *ρ* values. In Figure 5, the pink region is the S/D electrode, the blue region is the channel area, the arrow is E-field, and L is the length of electrode. Furthermore, *ρ* indicates the hemisphere radius, thus, a long *ρ* represents spherical corner shape at the channel/electrode boundary and a short *ρ* represents perpendicular corner shape. In Figure 5a, the device with circular corners shows identical color (i.e., identical magnitude of E-field) along the channel region. However, when the *ρ* is shortened and the corner becomes stiff, E-field is concentrated on the corner region and its magnitude is increased in Figure 5b. Furthermore, when *ρ* becomes zero as shown in Figure 5c, the E-field is more sharply focused on the corner than in Figure 5b. To quantitatively confirm its effect, enlarged images of simulation and E-field values along the channel toward the x-axis direction were presented in Figure 6. Figure 6a shows an enlarged one-side corner of the structure from Figure 5a. To closely see the difference of E-field between the corner and the common region, scanning areas are divided into three sections. For all of the sections, Figure 6a,b exhibit a relatively uniform distribution of E-field from 8 to 13 kV/cm along the channel. (The data of E-field = 0 indicates the measuring point is in the metal electrode region.) However, in Figure 6c,d, a highly enhanced E-field at Section 1 was shown with a value of 22 kV/cm at both corners. And for Section 2 and Section 3, which are slightly separate from the corner, the E-field is analogous to results from Figure 6a,b, indicating E-field is concentrated at the corner region. In the case of a device structure with *ρ* = 0, as shown in Figure 6e,f, E-field at Section 1 shows a remarkable increase with the value of 45 kV/cm at both corners, which is four-times larger than it is at Section 3. This result indicates the perpendicular shape of the corner induces more E-field enhancement than the spherical shape, thus, the E-field enhancement is directly associated with value of *ρ* For the confirmation of *ρ* dependency, E-field at Section 1 depending on various *ρ* was extracted in Figure 7. Similarily, the results of Figure 6 and Figure 7a show the two highest E-field peak at both corners. Its maximum value at the corner was gradually increased as *ρ* decreased. The detailed extracted E-field values depending on *ρ* were presented in Figure 7b. This result can be meaningfully when combined with results of bias temperature stress instability. In the device structure, *ρ* is calculated as 1.18 μm for zigzag-type a-IGZO TFTs and calculated as 3.09 μm for U-type a-IGZO TFTs, thus, it is expected to have larger E-field enhancement for the zigzag-type a-IGZO TFTs than the U-type device. Since zigzag-type a-IGZO TFTs show a larger *V_th_* shift than U-type device in Figure 4 and Figure 5, this expectation from TCAD simulation is well matched with experimental results. All of the results in this section substantiate the presence of E-field enhancement at the corner region of the channel, and its variation depending on hemisphere radius *ρ*.

## 4. Conclusions

In this study, we have investigated channel shape effects on device reliability for a-IGZO TFTs. From the transfer characteristics in linear and saturation regime, the S/D asymmetry effects were hard to be observed due to large transfer length (L_T_) compared with the width of gate electrodes associated with full gate overlapped structures. In parallel, regardless of channel shape in a-IGZO TFTs, vertical E-field stress on bias temperature stress instability turned out to be analogous for all of the devices, whereas lateral E-field effects were significantly pronounced for zigzag-type a-IGZO TFTs. Time dependence of ∆*V_th_* in a-IGZO TFTs is well fitted with a stretched-exponential equation, which can be derived from the assumption that the origin of threshold voltage shift is attributed to the trapping of charges in the interface and bulk dielectric layers. Furthermore, E-field enhancement effects on the locally sharpened source and the drain edge of the zigzag-type a-IGZO TFT can cause huge degradation of device instability. For elucidation of E-field enhancement at the corner region and its variation depending on hemisphere radius *ρ*, TCAD simulation was used. The simulation results revealed the locally enhanced E-field at both the corner region and its correlation with *ρ* values. Furthermore, the simulated results were well matched with experimental device instability tests associated with channel shape. We expect that this work can be beneficial for the provision of practical guidelines of device design rules in terms of channel shape determination, minimization of parasitic capacitance, and their correlated effects on the enhancement of device reliability in a-IGZO TFTs.

## Figures and Tables

**Table 1:** Summary of ∆*V_th_* and trapping time extracted from stretched exponential equation for 3-types a-IGZO TFTs.

Stress Condition	Parameter	Channel Shape		
		Circular	U-Type	Zigzag
V_GS_ = 25 V, V_DS_ = 0.1 V	∆*V_th_*_,max_ (V)	3.32	3.06	3.32
	Trapping time, *τ* (s)	1.6 × 10^4^	2.8 × 10^4^	1.1 × 10^4^
V_GS_ = 15 V, V_DS_ = 15 V	∆*V_th_*_,max_ (V)	1.40	1.42	2.10
	Trapping time, *τ* (s)	2.0 × 10^5^	3.4 × 10^5^	5.5 × 10^4^
